# *PBX3* hypermethylation in peripheral blood leukocytes predicts better prognosis in colorectal cancer: A propensity score analysis

**DOI:** 10.1002/cam4.2321

**Published:** 2019-05-29

**Authors:** Hongru Sun, Hao Huang, Dapeng Li, Lei Zhang, Yuanyuan Zhang, Jing Xu, Ying Liu, Yupeng Liu, Yashuang Zhao

**Affiliations:** ^1^ Department of Epidemiology Public Health College, Harbin Medical University Harbin The People’s Republic of China

**Keywords:** colorectal cancer, DNA methylation, *PBX3*, peripheral blood leukocyte, predictive biomarker

## Abstract

**Objective:**

The significance of gene methylation in peripheral blood leukocytes (PBLs) for assessing cancer prognosis is poorly understood. Our purpose is to assess the association between *PBX3* methylation in PBLs and colorectal cancer (CRC) prognosis.

**Methods:**

A total of 369 CRC patients were followed up for up to 10 years in this cohort study. PBL* PBX3* methylation levels were determined by methylation‐sensitive high‐resolution melting. Cox regression models and Log‐rank tests were used to analyze the associations between *PBX3* methylation status and CRC prognosis with a propensity score (PS) method to control confounding biases.

**Results:**

In this study, we found that CRC patients with PBL *PBX3* hypermethylation status had a better overall survival (OS) (hazard ratio [HR_PS‐adjusted_], 0.72 [95% CI, 0.52‐1.00]; *P = *0.049). Subgroup analyses showed that the beneficial effect of *PBX3* hypermethylation status on CRC 10‐years OS remained significant among UICC stage III patients ([HR_PS‐adjusted_], 0.60 [95% CI, 0.38 to 0.95]; *P* = 0.029) and colon cancer patients ([HR_PS‐adjusted_], 0.49 [95% CI, 0.26 to 0.92]; *P* = 0.027).

**Conclusion:**

PBL *PBX3* hypermethylation is positively associated with better prognosis of CRC, especially for the UICC stage III CRC patients and colon cancer patients.

## 1 INTRODUCTION

Colorectal cancer (CRC) is one of the most common gastrointestinal malignant tumors in the world and remains the third and fifth leading cause of cancer‐related deaths in the Western countries and Asian countries, respectively.^1^, ^2^ Currently, the most accurate means for assessing CRC patient prognosis require pathological staging of the tumor and the assessment of specific histological features of the tumor.^3^ However, the pathological staging is not accurate enough to predict the prognosis and recurrence and approximately 20%‐45% of those who undergo curative resection subsequently develop local tumor recurrence or metastasis at distant sites.^4^ Therefore, newer predictive biomarkers are urgently needed for accurate prediction of prognosis, reducing the rate of recurrence and thereby improving the overall survival (OS) of patients diagnosed with CRC. Furthermore, increasing evidence indicates that tumor arising from the colorectal tract can develop via a number of distinct pathways involving different combinations of genetic and epigenetic changes^5^, ^6^ including methylation.

Researchers have frequently focused on tumor tissues to explore the relationship between DNA methylation status and CRC prognosis. To date, several tumor tissue based DNA methylation biomarkers, including *CDKN2A*,^7^, ^8^, ^9^, ^10^, ^11^, ^12^, ^13^, ^14^, ^15^ *LINE‐1*,^16^, ^17^, ^18^, ^19^ *RET*,^20^ *KiSS1*,^21^ *MGMT*,^22^, ^23^ *EVL*,^24^ *IGFBP3*,^24^ *IGF2*,^25^ and *TFAP2E* 26 have been reported to be related with the prognosis of CRC. However, due to accessibility and high patient acceptance, peripheral blood DNA may be used as an ideal analyte for CRC biomarkers and peripheral blood is a readily available source of DNA that can be used to assess DNA methylation profiles. Recently, blood‐based circulation DNA methylation, such as *HTLF*,^27^, ^28^, ^29^ *HPP1* 27, 29 and *CDKN2A*,^30^ was detected as potential biomarker for prognosis of cancer. However, the content of circulation DNA in blood is limited and results in a larger amount of blood needed for detecting DNA methylation. It has been known that tumor initiation and progression do not develop as an isolated phenomenon in their target tissues, other organ systems including the immune system (such as peripheral blood leukocytes, PBLs) are also involved in tumor progression and prognosis. There have been several recent reports on peripheral blood‐based leukocyte DNA methylation biomarkers for various cancer risks, including breast,^31^ ovarian,^32^, ^33^ pancreatic,^34^ bladder,^35^ colorectal,^14^ and lung cancers.^36^ However, whether PBL DNA methylation can predict the prognosis of cancer including CRC has not been reported. Therefore, searching for the molecules that can serve as prognostic and predictive markers of CRC remains a priority.

Preleukemia transcription factor 3 (*PBX3*) is a member of the *PBX* family of three amino acid loop extension class homeodomain transcription factors, which are known to serve as cofactors for homeobox proteins and are physiologically involved in regulation of gene expression during embryonic development.^37^, ^38^ Some findings have documented that *PBX3* acts as an oncogenic gene in the progression of numerous cancer types.^39^, ^40^, ^41^, ^42^, ^43^, ^44^ It is worth noting that in CRC, *PBX3* has been associated with tumor progression and metastasis. Recent research reported that *PBX3* is a novel indicator of epithelial‐mesenchymal transition (EMT) in CRC, and a promising prognostic predictor that may aid in therapeutic decision‐making for patients with CRC.^45^ These data suggested oncogenic features of *PBX3* in CRC, but no previous study had evaluated whether *PBX3* methylation in PBL, as a noninvasive test, is a biomarker in CRC to predict prognosis.

For data analysis, we used not only univariate and traditional multivariate analysis but also a propensity score (PS) method, a newly proposed method that is used to reduce the likelihood of confounding bias when analyzing observational data from a cohort study in order to obtain results closer to a completely randomized control study.^46^ Therefore, in our 10‐year CRC cohort, we used PS‐based methods to comprehensively assess the effect of PBL *PBX3* methylation on CRC prognosis.

## 2 MATERIALS AND METHODS

### 2.1 Study design overview

**Figure 1:**
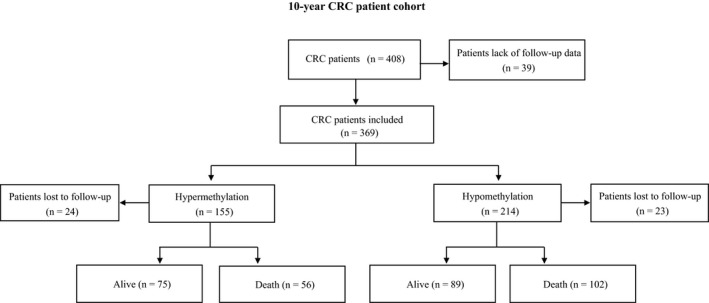
Flow chart of participant selection in the 10‐years CRC prognostic study

We compared the survival time between different PBL *PBX3* methylation status of CRC patients to derive the relationship between PBL *PBX3* methylation status and CRC patients' survival within this 10‐year follow‐up CRC cohort (Figure 1). In addition, we used PS methods to maximally control of the confounding bias and conducted sensitivity analyses to test the robustness of our findings.

### 2.2 10‐year cohort study for CRC prognosis analysis

The study population has been previously described^47^; briefly, in our initial cohort, a total of 521 eligible CRC patients with histological confirmation were recruited at the Third Affiliated Hospital of Harbin Medical University and the exclusion criteria included patients with inoperable metastatic CRC (n = 34), adenomatous polyposis coli (n = 27), a family history of CRC in first‐degree relatives (n = 16) according to the Amsterdam criteria 48 or patients who received any anticancer therapy before surgery (n = 15), unavailable blood sample (n = 19), or death within 30 days after surgery (n = 2). Then, 408 CRC patients were included in this analysis; however, 39 patients were further excluded due to the lack of follow‐up data. Thus, a total of 369 CRC patients were included in the final analysis and all subjects were operable stages I‐IV CRC patients. For each patient, demographic, clinicopathological, and treatment information were extracted from the electronic medical record system.

Ethics approval: All study participants provided written informed consent. This work has been approved by the Medical Ethics Committee of Harbin Medical University.

### 2.3 Follow‐up and outcomes

The primary outcome was OS from diagnosis to death and disease‐free survival (DFS) from diagnosis to disease recurrence or metastasis or death, whichever came first. Outcomes were observed via an established protocol during the follow‐up period through 15 March 2014. Patients were followed up postoperatively at a 6‐month interval for the first year and annually thereafter. We used a telephone follow‐up questionnaire to collect information on the date and cause of death of CRC patients. Among the 369 eligible CRC patients in the survival analysis, 158 patients died, 164 patients were still alive, and 47 patients were lost to follow‐up.

### 2.4 DNA extraction and bisulfite modification

DNA extraction and bisulfite modification were performed as previously described.^47^ Briefly, peripheral blood samples were centrifuged at 1600*g* for 10 minutes to separate the plasma and the buffy coats, and DNA was extracted from the buffy coats using a QIAamp DNA Blood Mini Kit (Qiagen, Hilden, Germany, Cat#51106) and then bisulfite‐modified using an EpiTect Plus DNA Bisulfite Kit (Qiagen, Cat#59826) according to the manufacturer's protocols. The bisulfite‐modified DNA sample was quantified using a NanoDrop 2000c bioanalyzer (Thermo‐Fisher, USA), diluted to a final concentration of 10 ng/µL and divided into aliquots for storage (−20°C).

### 2.5 Methylation analysis

We designed a methylation‐sensitive high‐resolution melting (MS‐HRM) assay for *PBX3* (GRCh37/hg19; chr9: 128651565‐128651668) using the Methprimer software.^49^ A set of methylation standards (100%, 10%, 5%, 1%, and 0% methylated DNA) were prepared by mixing commercially available methylated and unmethylated DNA (Zymo Research, Irvine, USA, Cat#D5014); these standards were used to semi‐quantitatively measure the DNA methylation status in the samples (Figure S1).

The MS‐HRM analysis was performed as previously described.^47^ Briefly, each PCR mixture consisted of a total volume of 10 µL containing 2 × LightCycler 480 High Resolution Melting Master Mix (Roche Applied Science, Mannheim, Germany, Cat#4909631001), 0.6 mmol/L MgCl_2_, 0.1 µmol/L of each primer (forward primer: CGGGATCGGAGGAAAGGGG; reverse primer: CGTCTACACACGTAAAAAACAAAA), and 1 µL (approximately 10 ng) of bisulfite‐modified template DNA. The PCR conditions were as follows: initial PCR activation (95°C for 15 minutes); 70 cycles of 3‐step amplification (95°C for 10 seconds, 58‐55°C (0.3°C/step) for 20 seconds, and 72°C for 20 seconds); and final extension (72°C for 10 minutes). A blank control (no‐template control) sample was included in each batch, and all reactions were performed in duplicate. A third trial was conducted for the samples that presented inconsistent results between the two trials. PCR amplification and MS‐HRM analyses were performed using the LightCycler 480 platform (Roche). After normalization of the melting curves using the Gene Scanning software (Roche), two investigators (HRS and HH) blinded to the outcomes assessed the MS‐HRM data. The discrepancies were resolved by discussion and consensus with another investigator (YPL).

### 2.6 Statistical analysis

Means and standard deviations or counts and frequencies are reported for the continuous or categorical variables, respectively. In the CRC prognosis analysis, the cut‐off point for *PBX3* methylation was ≥5% using the ROC method with the OS time as the dependent variable (0, less than median survival time; 1, longer than or equal to median survival time). According to this cut‐off point, CRC patients were categorized into *PBX3* hypomethylation and *PBX3* hypermethylation groups. A Kaplan‐Meier curve and the log‐rank test were then used to compare the OS and DFS between groups. Association between *PBX3* methylation and OS or DFS was estimated using the univariate and multivariate Cox regression models and Log‐rank tests and was reported as hazard ratios (HRs) and 95% CIs. Two‐sided statistical significance was defined as *P* < 0.05. The ROC analyses were performed with MedCalc version 12.6.1.0, and all other statistical analyses were performed with SPSS Statistics version 23.0 (IBM, Inc, USA).

To minimize group differences on covariates, we performed a PS‐based analysis. In the survival analysis, the PS was calculated with* PBX3* methylation as the dependent variable by using multivariate logistic regression models, which included clinicopathological characteristics (eg, tumor location, tumor size, UICC stage, pathological morphology type, tumor differentiation, adjuvant radio/chemotherapy, the level of serum carcinoembryonic antigen (CEA), and carbohydrate antigen 19‐9 (CA 19‐9) before surgery). To incorporate all patients in the analyses, we primarily employed the PS‐adjustment method. The differences in covariates between patients with hypermethylation versus hypomethylation of *PBX3* were compared with the standardized differences method, with a significant imbalance level of standardized difference ≥25%.

We performed several sensitivity analyses to explore the potential influence of different disease‐related factors on PBLs methylation status. Additionally, we established another PS model including composition of PBLs to observe the possible effects on our results of CRC prognosis. In addition, we compared the unadjusted effect estimates (HRs) with the adjusted effect estimates by using “confounding RR”. The confounding RR, which was defined as the ratio of the PS‐adjusted effect estimates and the minimally adjusted effect estimates, was calculated to evaluate the relative impact of PS adjustment for confounding factors. Finally, we performed subgroup analyses according to age (≥60 vs <60 years), gender (female vs male), BMI (≥24 vs <24), tumor location (colon or rectum), and tumor load (determined as UICC stage).

## 3 RESULTS

### 3.1 Characteristics of CRC patients

The basic demographic characteristics and clinicopathological features of the CRC patients in this 10‐year follow‐up cohort before and after PS adjustment are listed in Table 1.

**Table 1:** Baseline characteristics of CRC patients before and after propensity score adjustment

Characteristics	10‐year CRC cohort	*PBX3* Methylation in PBLs		Standardized Difference (%)	
		Hypomethylation (%)	Hypermethylation (%)	Before PS adjustment	After PS adjustment
Total number	369	214	155	−39.3	2.5
Age (years), Mean (SD)	58.49 (11.23)	58.31 (11.28)	58.72 (11.19)	−3.7	−6.5
<60	192 (52.0)	114 (53.3)	78 (50.3)		
≥60	177 (48.0)	100 (46.7)	77 (49.7)		
Gender				16.2	6.5
Male	219 (59.3)	120 (56.1)	99 (63.9)		
Female	150 (40.7)	94 (43.9)	56 (36.1)		
BMI (Kg/m^2^), Mean (SD)	23.39 (3.50)	23.24 (3.17)	23.84 (3.93)	11.0	6.4
<24.00	207 (56.1)	127 (59.3)	80 (51.6)		
≥24.00	162 (43.9)	87 (40.7)	75 (48.4)		
Tumor location				17.4	−6.2
Colon	129 (35.0)	82 (38.3)	47 (30.3)		
Rectum	240 (65.0)	132 (61.7)	108 (69.7)		
UICC stage					
I + II	196 (53.1)	112 (52.3)	84 (54.2)		
III	144 (39.0)	85 (39.7)	59 (38.1)	−3.4	4.8
IV	29 (7.9)	17 (8.0)	12 (7.7)	−0.8	−1.4
Pathological morphology				2.9	−1.8
Protruding type	241 (65.3)	141 (65.9)	100 (64.5)		
Ulcerative type	128 (36.7)	73 (34.1)	55 (35.5)		
Tumor differentiation				5.1	4.7
Well to moderate	66 (17.9)	40 (18.7)	26 (16.8)		
Poor	303 (82.1)	174 (81.3)	129 (83.2)		
Postoperative adjuvant chemotherapy				13.9	5.8
No	202 (54.7)	111 (51.9)	91 (58.7)		
Yes	167 (45.3)	103 (48.1)	64 (41.3)		
Postoperative adjuvant radiotherapy				8.4	6.6
No	344 (93.2)	198 (92.5)	146 (94.2)		
Yes	25 (6.8)	16 (7.5)	9 (5.8)		
Tumor size (mm) Median (IQR)	64 (27‐156)	64 (27‐150)	72 (30‐174)	−47.7	8.7
Preoperative CEA level Median (IQR)	7.30 (2.30‐16.05)	8.45 (2.10‐19.98)	5.50 (2.30‐15.20)	−2.9	2.1
Preoperative CA19‐9 level Median (IQR)	20.43 (9.67‐41.96)	22.12 (9.76‐60.09)	20.17 (9.57‐36.79)	5.5	2.7

Abbreviations: BMI = body mass index; CRC = colorectal cancer; CEA = carcinoembryonic antigen; CA19‐9 = carbohydrate antigen 19‐9; IQR = inter‐quartile range; PBL = peripheral blood leukocyte, PS = propensity score; SD = standard deviation.

### 3.2 PBL PBX3 methylation status predict survival risk in CRC cohort

**Figure 2:**
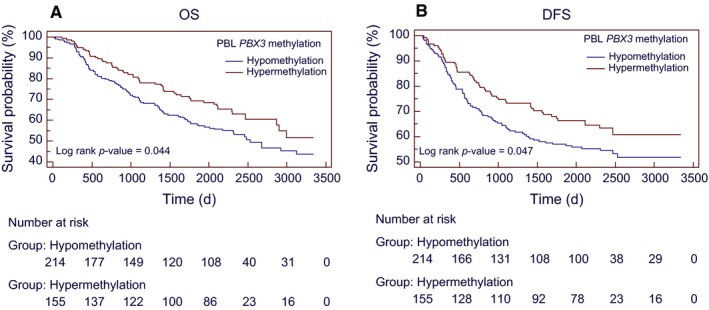
Kaplan‐Meier survival curves for the associations between PBL *PBX3* methylation and CRC prognosis. (A) 10‐years overall survival and (B) disease‐free survival according to PBL *PBX3* methylation status in overall CRC patients. Abbreviations: CRC = colorectal cancer; DFS = disease‐free survival; OS = overall survival; PBL = peripheral blood leukocyte; UICC = International Union Against Cancer

We investigated the potential roles of PBL *PBX3* methylation in predicting the prognosis of CRC in our 10‐year follow‐up CRC cohort. The median OS was 2238 days (IQR, 1107‐2393 days) in the *PBX3* hypermethylation group versus 2041 days (IQR, 834‐2431 days) in the hypomethylation group. The median DFS was 2103 days (95% CI, 817‐2388 days) in the* PBX3* hypermethylation group versus 1566 days (95% CI, 563‐2426 days) in the hypomethylation group. The 10‐year OS rate was 51.6% in the *PBX3* hypermethylation group versus 43.8% in the hypermethylation group. We found that in patients with PBL *PBX3* hypermethylation, there was a better than 10‐year OS ([HR_crude_]:0.72 [95% CI, 0.52‐0.99; *P* = 0.045]) and DFS ([HR_crude_]:0.70 [95% CI, 0.49‐0.99; *P* = 0.048]). The Kaplan‐Meier survival curves for OS and DFS are shown in Figure 2.

Table 2 shows the results of multivariate cox analysis of various factors for OS and DFS in all the patients. Multivariate analysis was performed for factors that showed significance in univariate analysis. After multivariate adjustment, we observed that the PBL *PBX3* hypermethylation in CRC patients was independently associated with a better 10‐year OS ([HR_Multivariate‐adjusted_]: 0.71 [95% CI, 0.51‐1.00; *P* = 0.049]), and marginally associated with a better DFS ([HR_Multivariate‐adjusted_]: 0.72 [95% CI, 0.51‐1.02; *P* = 0.06]).

**Table 2:** The results of univariate or multivariate cox regression models in this 10‐years CRC cohort study

Characteristics	Overall survival				Disease‐free survival			
	Univariate analysis HR (95% CI)	*P*	Multivariate analysis HR (95% CI)^b^	*P*	Univariate analysis HR (95% CI)	*P*	Multivariate analysis HR (95% CI)^c^	*P*
*PBL PBX3* (Hypermethylation vs Hypomethylation)	0.72 (0.52‐0.99)	0.045	0.71 (0.51‐1.00)	0.049	0.70 (0.49‐0.99)	0.048	0.72 (0.51‐1.02)	0.063
Age (≥60 vs <60)	0.95 (0.69‐1.29)	0.722	1.06 (0.77‐1.47)	0.710	0.90 (0.65‐1.24)	0.522	1.01 (0.77‐1.53)	0.629
Gender (Female vs Male)	1.10 (0.80‐1.51)	0.552	1.17 (0.84‐1.64)	0.359	1.15 (0.83‐1.60)	0.389	1.22 (0.86‐1.72)	0.260
BMI (≥24.0 vs <24.00)	0.98 (0.71‐1.33)	0.837	1.15 (0.83‐1.60)	0.391	0.80 (0.58‐1.12)	0.192	0.93 (0.66‐1.30)	0.650
UICC stages (III vs I + II)	3.30 (2.13‐4.32)	<0.0001^a^	2.49 (1.73‐3.60)	<0.0001	2.97 (2.05‐4.30)	<0.0001^a^	2.28 (1.55‐3.34)	<0.0001
UICC stages (IV vs I + II)	8.47 (5.21‐13.76)	<0.0001^a^	4.111 (2.35‐7.18)	<0.0001	7.27 (4.45‐11.87)	<0.0001^a^	3.38 (1.92‐5.93)	<0.0001
Pathological morphology (Ulcerative type vs Protruding type)	1.91 (1.40‐2.62)	<0.0001^a^	1.49 (1.07‐2.07)	0.018	2.01 (1.46‐2.78)	<0.0001^a^	1.41 (1.00‐1.99)	0.049
Tumor location (Rectum vs Colon)	1.15 (0.83‐1.61)	0.405			0.99 (0.84‐1.17)	0.901		
Tumor differentiation (Poor vs Well to moderate)	0.60 (0.41‐0.88)	0.008^a^	0.62 (0.42‐0.91)	0.016	0.52 (0.37‐0.75)	0.001^a^	0.57 (0.39‐0.85)	0.006
Postoperative adjuvant chemotherapy (Yes vs No)	0.99 (0.72‐1.35)	0.924			0.62 (0.45‐0.85)	0.003^a^	0.72 (0.50‐1.03)	0.068
Postoperative adjuvant radiotherapy (Yes vs No)	0.43 (0.26‐0.72)	0.001^a^	0.80 (0.45‐1.42)	0.437	0.36 (0.21‐0.60)	<0.0001^a^	0.71 (0.38‐1.32)	0.271
Preoperative CEA level (≥5 ng/mL vs <5 ng/mL)	1.91 (1.36‐2.67)	<0.0001^a^	0.98 (0.66‐1.45)	0.907	2.10 (1.48‐2.99)	<0.0001^a^	1.14 (0.76‐1.72)	0.516
Preoperative CA19‐9 level (≥37 U/mL vs <37 U/mL)	4.33 (3.43‐6.52)	<0.0001^a^	3.88 (2.66‐5.66)	<0.0001	4.06 (2.91‐5.66)	<0.0001^a^	3.0 (2.03‐4.43)	<0.0001
Tumor size (≥400 mm vs <400 mm)	1.49 (1.04‐2.13)	0.029^a^	1.47 (1.01‐2.14)	0.044	1.62 (1.12‐2.36)	0.012^a^	1.62 (1.09‐2.40)	0.017

Abbreviations: CRC = colorectal cancer; CI = confidence interval; CEA = carcinoembryonic antigen; CA 19‐9 = carbohydrate antigen 19‐9; BMI = body mass index; HR = hazard ratio; PBL = peripheral blood leukocyte; UICC = International Union Against Cancer.

a Characteristics with statistically significant *P* values were included in the multivariate model.

b Multivariate adjusted factors in overall survival: age, gender, BMI, UICC stages, pathological morphology, tumor differentiation, postoperative adjuvant radiotherapy, preoperative CEA, preoperative CA19‐9, tumor size.

c Multivariate adjusted factors in disease‐free survival: age, gender, BMI, UICC stages, pathological morphology, tumor differentiation, postoperative adjuvant radiotherapy, postoperative adjuvant chemotherapy, preoperative CEA, preoperative CA19‐9, tumor size.

### 3.3 Propensity score‐adjusted analysis for the associations between PBL PBX3 methylation and CRC prognosis

**Figure 3:**
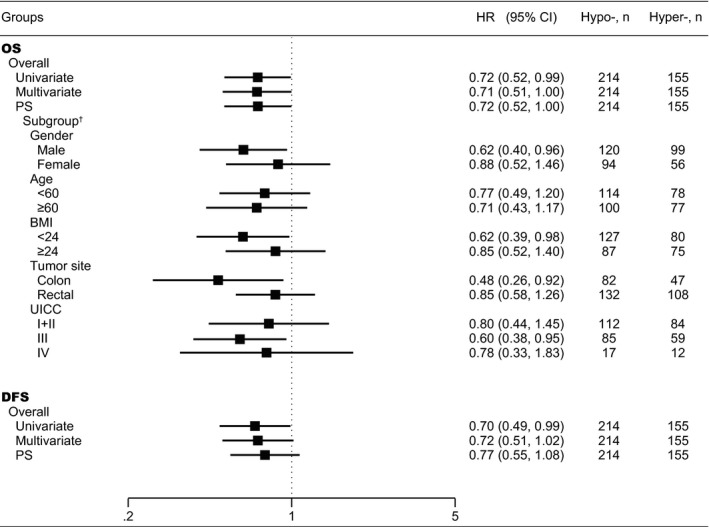
Associations between PBL *PBX3* methylation and CRC prognosis in 10‐years OS and DFS. † Subgroups HR values are the effect estimates adjusted by propensity score. Abbreviations: BMI = body mass index; CRC = colorectal cancer; CI = confidence interval; DFS = disease‐free survival; HR = hazard ratio; Hyper‐=*PBX3* hypermethylation; Hypo‐=*PBX3* hypomethylation; OS = overall survival; PBL = peripheral blood leukocyte; UICC = International Union Against Cancer

To be more conservative and minimize confounding biases, we further performed a PS‐based analysis and still found a significant association of *PBX3* hypermethylation with a better OS ([HR_PS‐adjusted_], 0.72 [95% CI, 0.52‐1.00]; *P = *0.049) but not significantly associated with DFS ([HR_PS‐adjusted_], 0.77 [95% CI, 0.55‐1.08]; *P = *0.132) (Figure 3).

**Figure 4:**
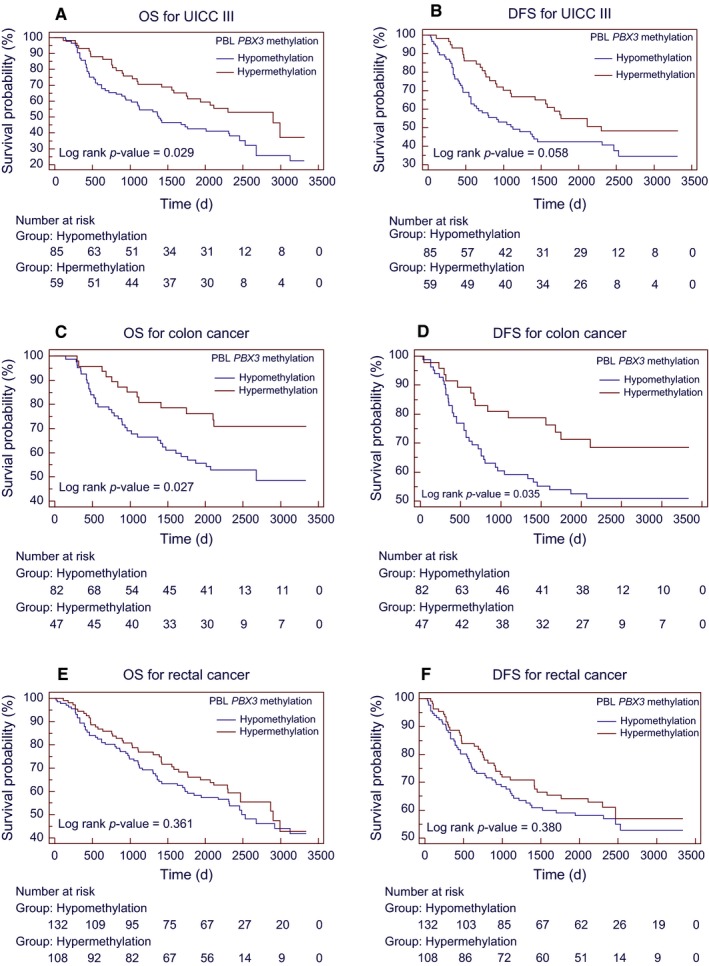
Kaplan‐Meier survival curves for OS or DFS according to PBL *PBX3* methylation status among CRC patients with UICC stage III (A, B), colon cancer (C, D) and rectal cancer (E, F). Abbreviations: CRC = colorectal cancer; DFS = disease‐free survival; OS = overall survival; PBL = peripheral blood leukocyte; UICC = International Union Against Cancer

Based on subgroup analyses, we found that the association of *PBX3* hypermethylation with a better OS was significant only among colon cancer, UICC stage III cancer, male, or normal weight patients, whereas the effect estimates did not reach statistical significance among rectal cancer, stage I + II and IV cancer, female, and overweight or obese patients. The results of subgroup analyses adjusted by PS are shown in Figure 3. The Kaplan‐Meier survival curves in UICC stages III CRC, colon or rectum cancers are shown in Figure 4.

### 3.4 Sensitivity analysis

We compared PBL *PBX3* methylation status among subgroups of all the characteristics and clinicopathological features in 10‐year CRC cohort prognosis study. Our data did not indicate elevated methylation frequency with respect to the factors shown in Table 1 in this study (all *P*‐value > 0.1) (Table S1). In addition, we collected the clinical record of leukocyte counts and composition of PBLs and included these data in the PS model in the prognosis analysis ([HR_PBL_]: 0.72 [95% CI, 0.52 to 1.01]; *P* = 0.055) (Table S2). Notably, we found a marginally significant relationship between PBL *PBX3* methylation levels with leukocyte count and percentage composition of leukocyte subfractions and CRC prognosis. By using “confounding RR”, we found no heterogeneity between them. Generally, the unadjusted HRs for the association between *PBX3* hypermethylation and CRC prognosis were attenuated compared to those observed in the PS‐adjustment dataset. However, the attenuation was not statistically significant (Figure S2).

## 4 DISCUSSION

In the present work, we found a positive association between PBL *PBX3* methylation and CRC prognosis in a 10‐year cohort study and confirmed the main findings in a PS method‐based analyses study. *PBX3* methylation in PBLs was an epigenetic alteration detectable in accessible, nondiseased tissue that predicts the prognosis of CRC. This is the first study with a long follow‐up and relatively large sample size to address the prognostic association of *PBX3* methylation in PBLs among CRC patients.

Our findings demonstrated that patients with PBL *PBX3* hypermethylation had significantly favorable 10‐year OS and DFS than patients with PBL *PBX3* hypomethylation. Moreover, in our study, we included 13 clinical variables in PS models of CRC prognosis analysis. After PS adjustment, there were no significant baseline characteristic differences between groups (≤25%). Generally, by using PS methods, we can include many covariates in the PS model, and accordingly substantially limit the number of covariates used in the final analysis. Our results on multivariate adjusted and PS‐adjusted cox proportional hazard model analysis indicated that PBL *PBX3* hypermethylation was an independent prognostic biomarker for 10‐year OS simultaneously. However, we found that the association between PBL *PBX3* methylation and DFS did not reach statistical significance in PS‐adjusted model. This may be due to the limitation of our sample size and the conservative nature of the PS method. However, we can still see the trend in the Kaplan‐Meier survival curve of DFS. Further large cohort studies are required to validate this issue.

To ensure the validity of our findings, we performed not only PS‐based analyses but also extensive sensitivity analyses to assess the robustness of our findings (Table S1). Assessing the potential influence of the basic demographic characteristics and clinicopathological features on methylation status, we found no effect of any characteristics and clinicopathological features in our patients (such as, UICC stage, preoperative CEA level, preoperative CA19‐9 level, and composition of PBLs). The confounding RR can assess how strong the adjusted confounds are or an unmeasured confound that would have an impact on the observed associations. Based on these sensitivity analyses, our results were unlikely to be substantially impacted by both the adjusted confounds included in the PS models and a potential residual confound. Of importance, the fraction of circulating tumor cells is estimated to be less than 1 millionth versus PBLs detected in the circulation,^50^, ^51^ and the concentration of free tumor DNA in plasma is far lower than that observed in tissue and blood cells, so the possibility that our results may have been influenced by tumor DNA contamination seems negligible. Furthermore, in the subgroup analyses of CRC prognosis, we found that *PBX3* hypermethylation was associated with better 10‐years OS in the male, normal weight, colon, and UICC stage III subgroups (Figure 3). At present, a research result shows that *PBX3* was required for the complete EMT phenotype in colon cancer cells.^45^ But the reasons for these phenomena in PBLs were still unclear and need to be validated in future studies.

Recent mechanism researches indicated that *PBX3* expressed in tumor cells with high WNT activity undergoing EMT as a new indicator that is associated with poor prognosis in CRC and *PBX3* mRNA expression was also highly significantly associated with poor outcome.^45^ Other research found that let‐7c serves as a tumor metastasis suppressor by inhibiting *PBX3* mRNA expression.^52^ Therefore, we further analyzed the relationship between *PBX3* mRNA expression in tumor tissue samples and CRC prognosis in the TCGA dataset and confirmed the association between *PBX3* higher expression and a poor CRC prognosis ([HR_OS_]: 1.44 [95% CI, 1.03 to 2.01]; *P* = 0.034). Additionally, it is interesting that *PBX3* hypermethylation in the transcription start site (TSS) region was significantly associated with a better OS ([HR_OS_]: 0.60 [95% CI, 0.39 to 0.93]; *P* = 0.022). Through the Kaplan‐Meier survival curves of TCGA, we could clearly see the trend, that is, both the *PBX3* hypermethylation in the TSS region and lower mRNA expression levels of *PBX3* were significantly associated with a better CRC prognosis (Figure S3). These were not only consistent with the findings in our PBL collections but also confirmed results from previous studies that suggested an association of *PBX3* mRNA expression and poor patient survival. These findings suggested that *PBX3* methylation may affect the expression of mRNA and plays an important role in the progression in prognosis of CRC. However, the regulation of *PBX3* methylation in CRC and its contribution to tumor progression are still in need of further study. However, the functional consequence of the differences in methylation between individuals with and without *PBX3* hypermethylation in PBL was incompletely understood.

Our results may have several strengths and clinical significances. First, PBL *PBX3* hypermethylation was significantly associated with a better OS and obviously correlated with a longer OS time in a collection of 144 stage III CRC patients or in 129 patients with colon cancer, while this was independent of other core clinical variables. PBL *PBX3* methylation as a DNA‐based noninvasive blood test which could help to identify follow‐up CRC patients at higher risk for disease recurrence and prognosis. Second, metastatic CRC patients generally have obvious clinical characteristics and poor prognosis,^53^ and approximately 30%‐40% of UICC stage III patients will still develop tumor recurrence and a poor prognosis. Therefore, our findings provide a good direction for increasing clinical attention in patients with stage III. Third, the PS method is a powerful statistical tool to control for confounding variables and is often more practical and statistically more efficient than those conventional strategies including matching on covariates, stratified analyses, or multivariate statistical methods.^46^ Nonetheless, it will still be required to determine the true prognostic biomarker potential of this noninvasive blood test in routine clinical practice by robust multicenter validation studies in prospectively recruited patients.

Our research also has several limitations. The main limitation of our study is that we cannot definitively determine whether the differential expression of DNA methylation in PBLs is a response of the hematopoietic systems to the presence of the malignant tumor which affects the immune system or in some way allow for or potentiate the growth of the tumor. At the same time, we have hypothesized that these differences may represent a directed alteration and that by looking at the gene whose differential methylation region was associated with the prognosis of CRC, we may be able to better define how these pathologic processes are influencing methylation status. The functions and mechanisms of DNA methylation in PBLs affecting the prognosis of CRC need deeper research in the future. Second, we had evaluated an important concern about DNA methylation in PBL subpopulations that may affect the methylation signature of an individual, since we performed another PS model induced the information of the PBL subpopulations. This makes our results marginally associated with OS. We believed that the reason for minor change on our result was also due to the conservative nature of the PS. At the same time, a recently published study showed that the difference in leukocyte subpopulations was unlikely to interfere with the results of PBL‐derived DNA methylation suggesting that the effect of leukocyte count and subpopulations on our results may be insignificant.^54^ Third, because of the observational nature of our study, unmeasured confounding variables may have influenced the reported associations, but our PS analyses and sensitivity analyses suggest that substantial confounding is highly unlikely. Lastly, although our results are not at the level required for immediate predictive utility, they do point, along with a small but growing number of other studies of other solid tumors, to the tremendous clinical potential of epigenetic profiling of PBL DNA.

## 5 CONCLUSION

Our findings in PBL of CRC patients with 10‐year follow‐up data suggest that *PBX3* hypermethylation is an independent predictor to better OS of CRC patients, especially in patients with stage III or colon cancer.

## CONFLICT OF INTEREST

The authors declare no potential conflicts of interest.

## DISCLOSURES

All authors confirm that no part of this study is under consideration for submission elsewhere; has not been published or posted elsewhere; and will not be posted or published elsewhere.

## AUTHOR CONTRIBUTION

YSZ and YPL had full access to all the data in the study and take responsibility for the integrity of the data and the accuracy of the data analysis. YSZ and YPL contributed to study conception and design. HRS, YPL, HH, LZ, YYZ, JX, and YL contributed to DNA preparation and bisulfite modification. HRS and HH contributed to MS‐HRM. YPL, HRS, HH, LZ, and YYZ contributed to collection and assembly of data. YSZ, YPL, HRS, and DPL contributed to the analysis and interpretation of data. HH and JX contributed to sample collection. HRS, and LYP contributed to the drafting of the initial versions of the manuscript. YSZ, HRS, and YPL contributed to revise the manuscript until submit. All authors contributed to the review and final approval of the manuscript. YSZ and YPL were responsible for study supervision.
