# Supplementary material for: PBX3 hypermethylation in peripheral blood leukocytes predicts better prognosis in colorectal cancer: A propensity score analysis
Source: Cancer Med. 2019 May 29;8(8):4001–11. doi: 10.1002/cam4.2321 (PMC6639175; doi:10.1002/cam4.2321)

PBX3 Hypermethylation in Peripheral Blood Leukocytes Predicts Better Prognosis in Colorectal Cancer: a Propensity Score Analysis.

Hongru Sun, Hao Huang, Dapeng Li, Zhang Lei, Zhang Yuanyuan, Xu Jing, Liu Ying, Yupeng Liu#, Yashuang Zhao#

Supplemental Material

Correspondence to: Dr. Yashuang Zhao
Department of Epidemiology, Public Health College
Harbin Medical University
Harbin 150081, P. R. China
Tel: +86 (0) 451 8750 2823
Fax: +86 (0) 451 8750 2885
E-mail: zhao_yashuang@263.net

Supplementary Table 1. Sensitivity analyses for associations of *PBX3* methylation status with characteristics and clinicopathological features.

Characteristics	*PBX3* methylation in PBLs		*P*-value
	Hypomethylation	Hypermethylation	
**UICC stage**			0.939
I+II	112	84	
III	85	59	
IV	17	12	
**Preoperative CEA level**			0.509
<5 ng/ml	92	72	
≥5 ng/ml	122	83	
**Preoperative CA19-9 level**			0.342
<37 U/ml	153	117	
≥37 U/ml	61	38	
PBL counts, median (IQR)	6.50 (5.50-8.03)	6.60 (5.80-7.60)	0.861
Lymphocytes percentage	26.65 (20.75-31.68)	27.80 (21.80-34.20)	0.092
Neutrophil percentage	65.05 (58.93-71.70)	63.30 (55.60-69.90)	0.115
Monocyte percentage	6.31 (4.30-8.32)	6.18 (4.20-8.00)	0.833
Eosinophils percentage	0.80 (0.30-1.76)	0.92 (0.40-1.94)	0.187
Basophil percentage	0.20 (0.08-0.62)	0.30 (0.10-0.74)	0.197

Abbreviations: BMI=body mass index; CRC=colorectal cancer; CEA=carcinoembryonic antigen; CA19-9=carbohydrate antigen 19-9; IQR=inter-quartile range; PBL=peripheral blood leukocyte, SD=standard deviation.

Supplementary Table 2. Associations between PBL *PBX3* methylation and CRC prognosis in 10-years OS and DFS adjusted by PS including composition of PBLs.

Gene<br>Methylation Status	*PBX3* Methylation<br>Hypomethylation	 Hypermethylation	HR† (95% CI)	*P*-value
**OS**	214	155	0.72 (0.52-1.01)	0.055
**Subgroup**				
**Gender**				
Male	120	99	0.61 (0.40-0.94)	0.025
Female	94	56	0.95 (0.56-1.60)	0.840
**Age**				
<60	114	78	0.76 (0.49-1.20)	0.231
≥60	100	77	0.75 (0.46-1.24)	0.267
**BMI**				
<24	127	80	0.61 (0.24-0.88)	0.036
≥24	87	75	0.89 (0.54-1.47)	0.642
**Tumor site**				
Colon	82	47	0.46 (0.24-0.88)	0.019
Rectal	132	108	0.86 (0.59-1.28)	0.463
**UICC**				
I+II	112	84	0.80 (0.44-1.45)	0.460
III	85	59	0.59 (0.37-0.94)	0.027
IV	17	12	1.02 (0.44-1.45)	0.460
**DFS**	214	155	0.78 (0.55-1.09)	0.146

†Subgroups HR values are the effect estimates adjusted by propensity score including composition of PBLs.

Abbreviations: BMI=body mass index; CRC=colorectal cancer; CI=confidence interval; DFS=disease-free survival; HR=hazard ratio; OS=overall survival; PBL=peripheral blood leukocyte; UICC=International Union Against Cancer.

Supplementary Figure 1. High-resolution melting curves for PBL *PBX3* methylation standards. (A) Normalization plots; (B) Melting peak plots.

(A) Normalization plots

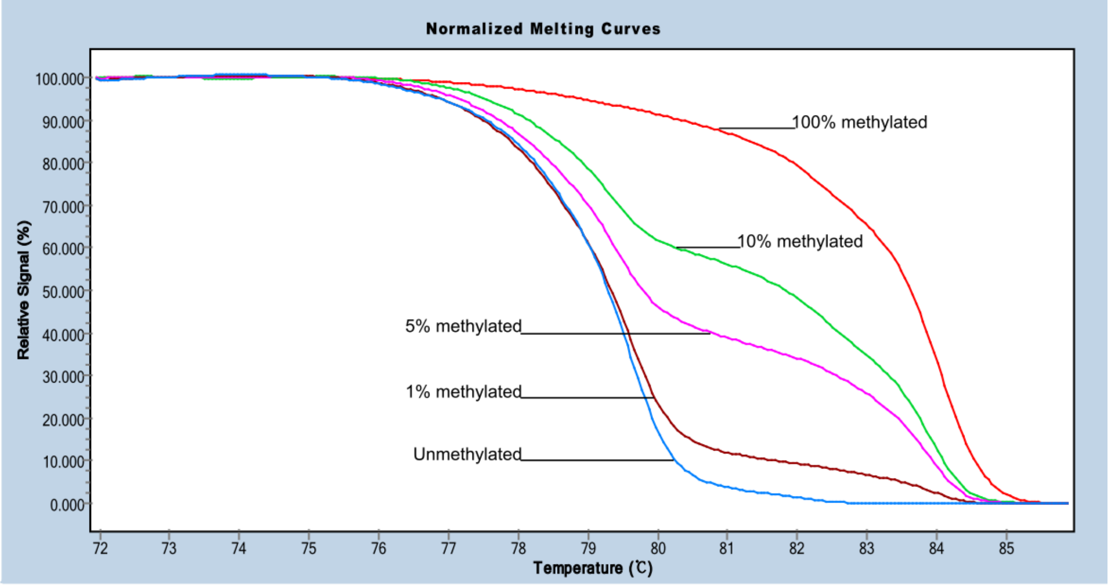

(B) Melting peak plots

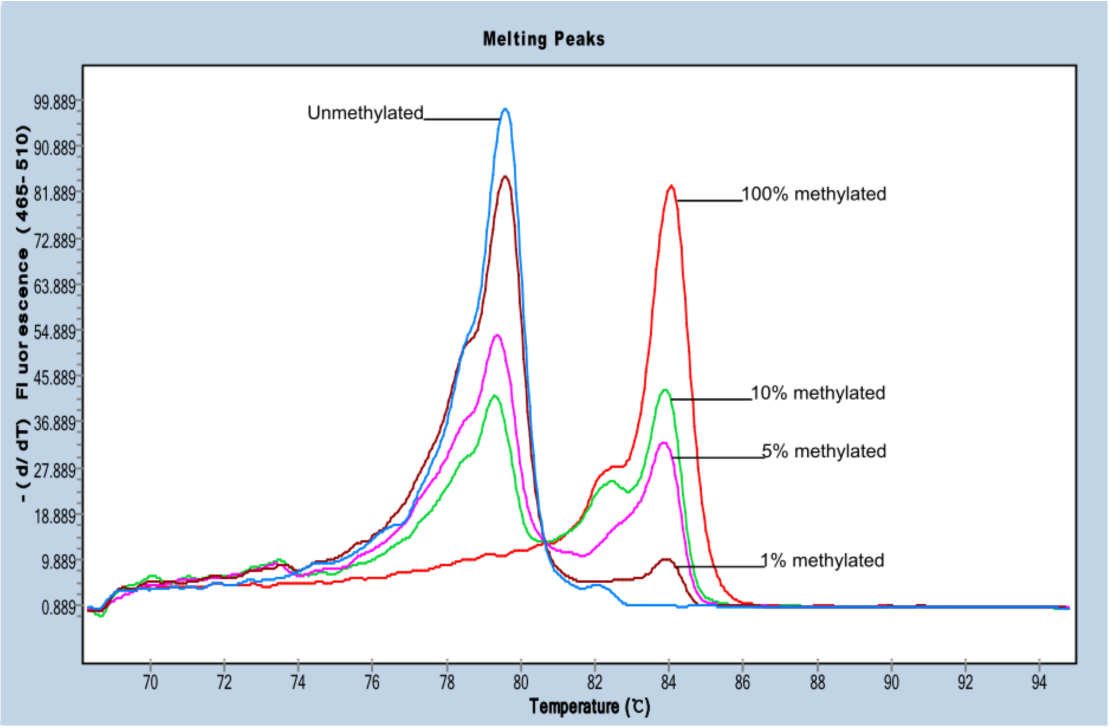

Supplementary Figure 2. Comparison of the unadjusted and the PS-adjusted effect estimates by using "confounding RR".

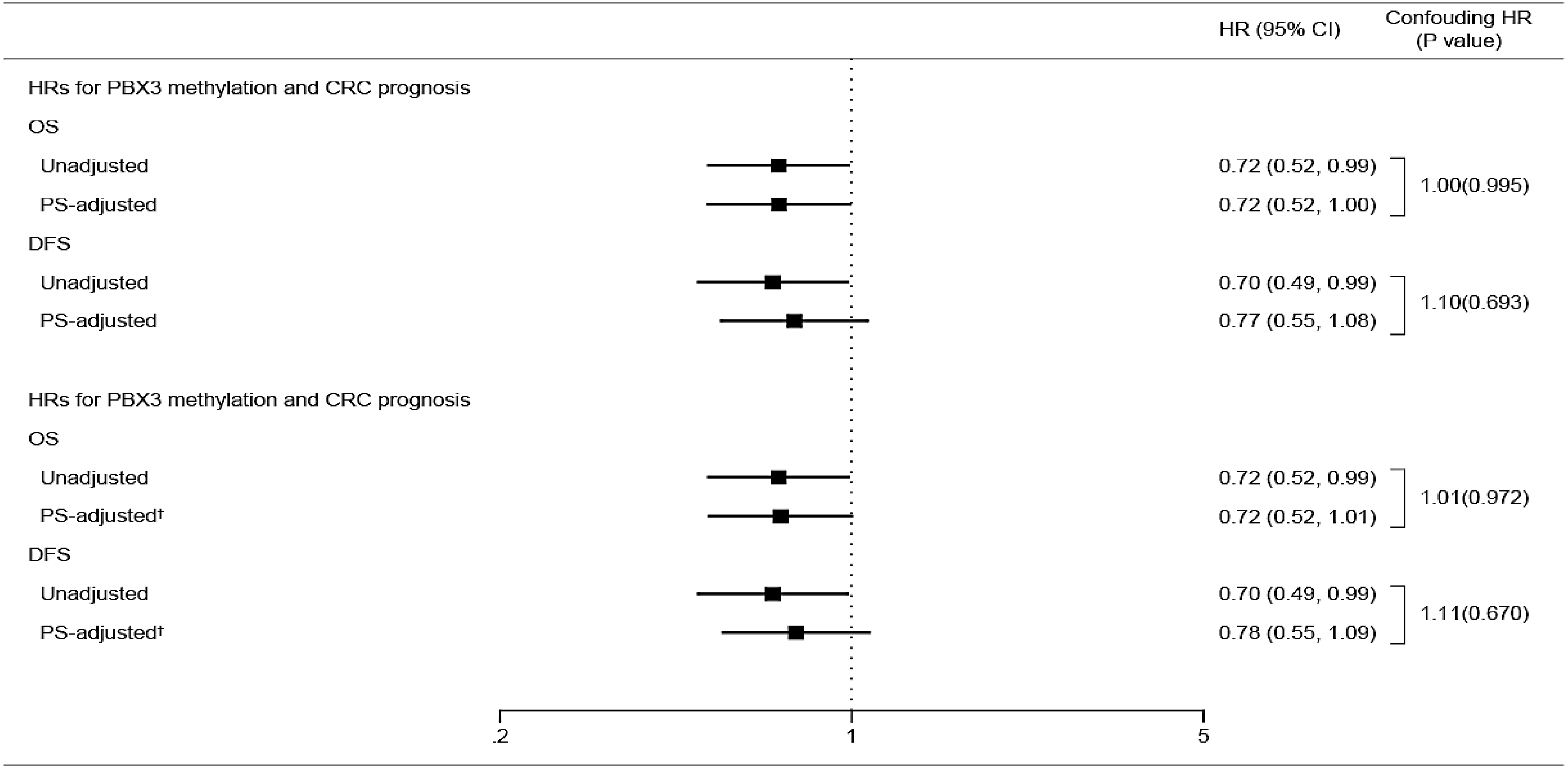

Note: The confounding RR, which was defined as the ratio of the PS-adjusted effect estimates and the minimally adjusted effect estimates, was calculated to evaluate the relative impact of PS-adjustment for confounding factors.

†PS-adjusted including composition of PBLs

Abbreviations: CRC=colorectal cancer; DFS=disease-free survival; HR=hazard ratio; OR=odds ratio; OS=overall survival; PS=propensity score; PBL=peripheral blood leukocyte.

Supplementary Figure 3. Kaplan-Meier survival curves of OS according to *PBX3* mRNA expression status among CRC patients in TCGA data (A); OS according to *PBX3* methylation status among CRC patients in TCGA data (B).

(A)

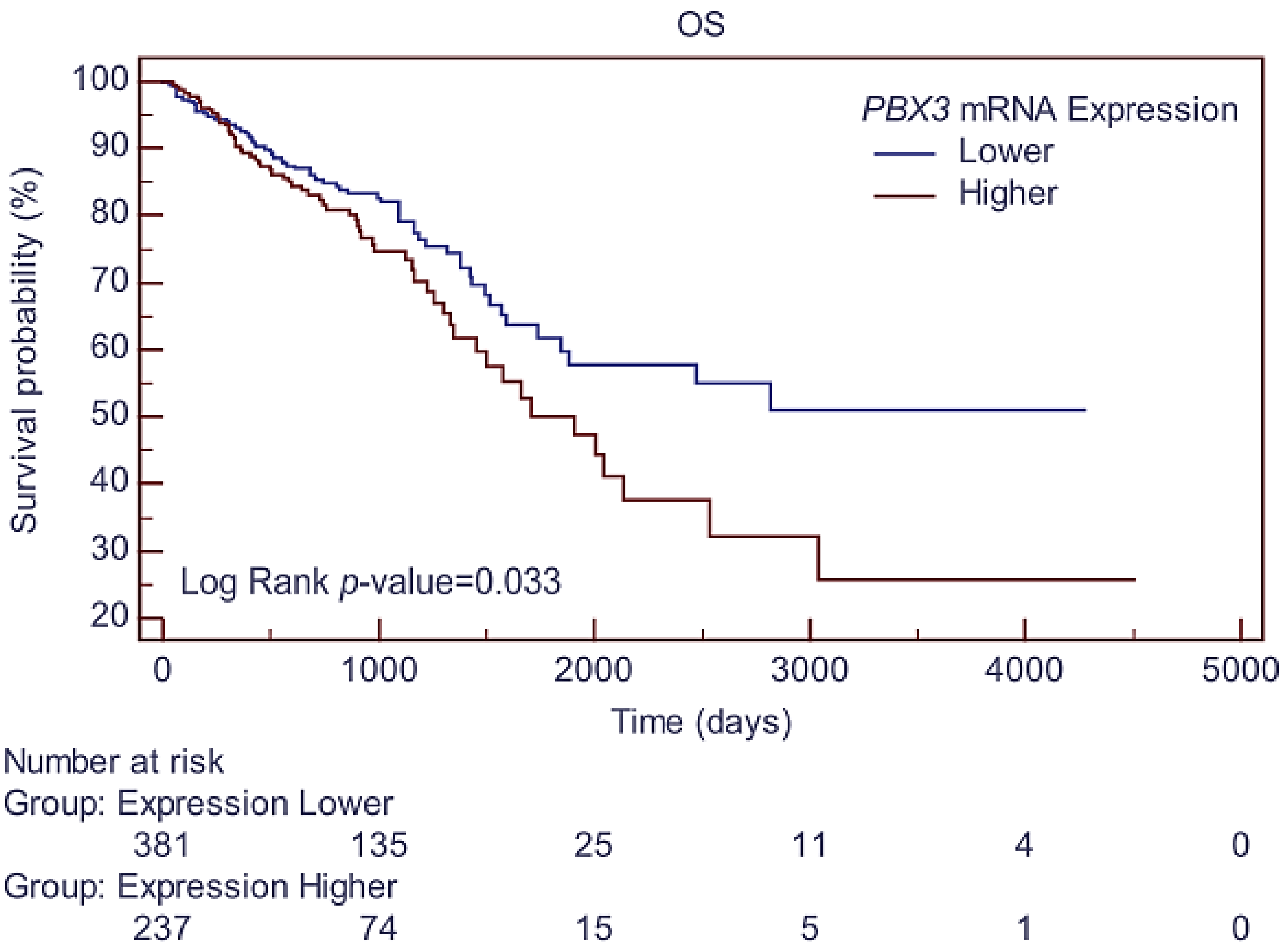

(B)

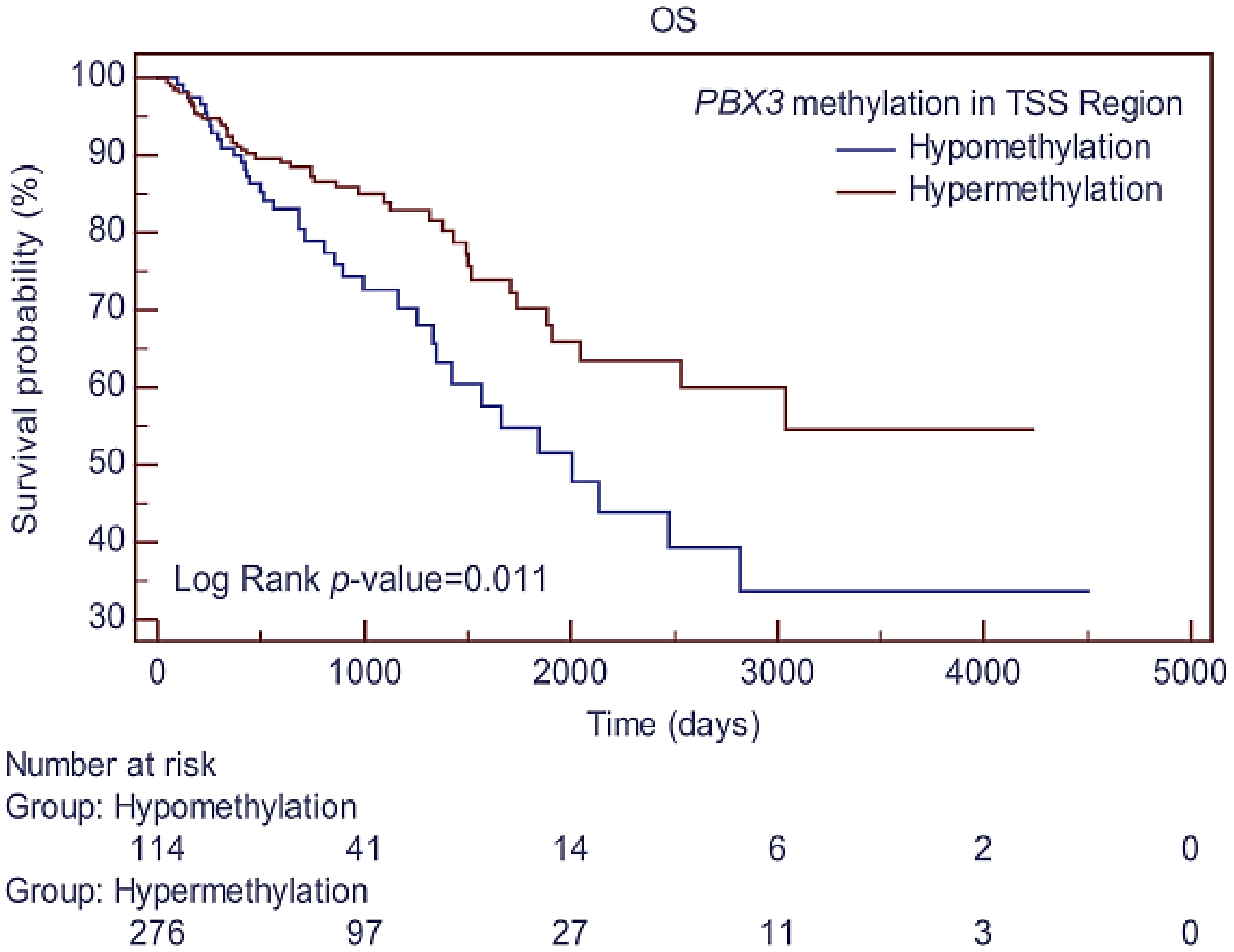

Supplement: Supplementary file 1 [file CAM4-8-4001-s001.pdf]
